# A new chapter for the Journal of Medicine and Life in 2021

**DOI:** 10.25122/jml-2021-1001

**Published:** 2021

**Authors:** Dragos Cretoiu, Stefan Strilciuc, Dafin Muresanu

**Affiliations:** 1. “Carol Davila” University of Medicine and Pharmacy, Bucharest, Romania; 2. Department of Neurosciences, “Iuliu Hatieganu” University of Medicine and Pharmacy, Cluj-Napoca, Romania; 3. “RoNeuro” Institute for Neurological Research and Diagnostic, Cluj-Napoca, Romania

As COVID-19 gradually shifts to an endemic state, stakeholders throughout academia have started settling in with an emerging “new normal” paradigm for science, dominated by the virtual environment. Journals make no exception to this phenomenon and are now striving to keep up with a myriad of developments in many fields, partly due to enhanced global focus on science. Despite pandemic-related hardship, the Journal of Medicine and Life (JML) has continued to grow, receiving significantly more and higher quality submissions from researchers worldwide.

To accommodate the heightened interest for dissemination in JML, as well as a strong incentive coming from the academic community to promote content at better standards in general, our editorial board and ownership has committed to an ambitious reform pathway, which we hope will rise JML to a top-tier publication in the coming years. In this editorial, we are uncovering exciting developments coming into effect starting this first issue of 2021, hoping to ignite interest in the journal and invest in working with us to disseminate your work.

First and foremost, we would like to present some information about this publication to readers who are not aware of the background of JML. It was founded as a peer-reviewed journal in 2008 and has published over 1.200 pieces of original work addressing topics from various fields of medicine and life sciences, including original research, systematic reviews, special reports, case presentations, major medical breakthroughs, and expert communications. We have also been continuously advocating for an educational and community-building environment by ensuring designated sections that inform our readers about exciting international congresses, teaching courses, and other relevant academic events.

Despite having reached some important indexing milestones (i.e., PubMed, ProQuest, Index Copernicus, EBSCO Host, CNCSIS B+), we are now looking to expand our reach toward new horizons by preparing to apply for inclusion in Clarivate Analytics Web of Science databases.

In doing so, we have started a profound reorganization of the journal, addressing several important facets. The most striking change is our brand-new visual identity, which had already been rolled out within our website during last year. Starting this first issue of 2021, we have also given our print edition a modern, fresh look, matching our authors’ immersive content. To support efforts on this front and improve the overall quality of journal output, our editorial team has also been expanded with several consultants (i.e., illustrators, statisticians).

Moreover, we have decided to increase our publication frequency from four to six issues per year (bi-monthly). We hope this will attract potential authors looking for a shorter average duration from submission to publication. The Journal of Medicine and Life will continue to be provided under the Creative Commons license, supported by our fees, which have now been consolidated under a single article processing charge, change which we hope will incentivize high-quality submissions to the journal. A crucial planned area of intervention is also consolidating and updating our editorial board, which will develop a brand-new, attractive editorial process that will enable the journal to reach the highest impact in the coming years.

Finally, we would like to highlight our recent partnership with NeurotechEU – the European University of Brain and Technology (https://www.theneurotech.eu). The NeurotechEU Alliance, bringing together eight leading universities and 250+ partners from all regions of Europe, aims to build a trans-European network of excellence in brain research and technologies to increase the competitiveness of European education, research, economy and society. In the upcoming 2021 issues, we plan to showcase our partners’ contributions to the broader topic of widening access and participation in the higher education arena.

We thank our readership and authorship for your most valuable contribution in supporting us come thus far and genuinely hope you will consider joining our efforts to bring this new chapter of JML’s existence to its full potential. If you are interested in joining our efforts in any way possible, please write us a letter at office@medandlife.org.

